# Collecting duct carcinoma of the kidney: Imaging observations of a rare tumor

**DOI:** 10.3892/ol.2013.1739

**Published:** 2013-12-06

**Authors:** YUXIAO HU, GUANG-MING LU, KAI LI, LONG-JIANG ZHANG, HONG ZHU

**Affiliations:** 1 Department of Nuclear Medicine, Jinling Hospital, Clinical School of Medical College, Nanjing University, Nanjing, Jiangsu 210002, P.R. China; 2 Department of Medical Imaging, Jinling Hospital, Clinical School of Medical College, Nanjing University, Nanjing, Jiangsu 210002, P.R. China; 3 Department of Pharmacology, Soochow University, Suzhou, Jiangsu 215123, P.R. China

**Keywords:** collecting duct carcinoma, computed tomography, kidney, positron emission tomography

## Abstract

Collecting duct carcinoma (CDC) is a rare type of renal neoplasm. Early diagnosis is possibly the only factor leading to a prolonged survival for patients with CDC. The purpose of the present study was to characterize the imaging features of CDC and improve its diagnosis. Radiological data of six patients were retrospectively reviewed by three experienced radiologists, including six cases examined with non-contrast computed tomography (CT) scans, five with contrast-enhanced CT scans, one with magnetic resonance urography, one with renal dynamic imaging and two with conventional whole-body ^18^F-fluorodeoxyglucose (FDG) positron emission tomography (PET)/CT scans. All patients were pathologically confirmed with CDC. In total, seven tumors were detected in the six cases, with a mean size of 5.3 cm. Of the tumors, two were solid and the rest were complex solid and cystic. In addition, six tumors were located in medullary areas and only one tumor was found in the cortical location. Cystic components were observed in five tumors. Weak enhancements were observed in all six tumors examined with contrast-enhanced CT, and heterogeneous enhancements were also observed in the majority of these tumors with the exception of one tumor. Infiltrative growth and expansible growth were found in five and two tumors, respectively. Metastatic lesions were detected in all six patients. On MR urography, the involved kidney exhibited similar imaging observations to the CT scan. Renal dynamic imaging revealed a decreased renal function in the involved kidney and an increased renal function in the contralateral kidney. On PET/CT imaging, a marked uptake of ^18^F-FDG was found in primary and metastatic lesions. The results of the present study indicated that medullary location, weak and heterogeneous enhancement, infiltrative growth, damage of renal function in the involved kidney and a marked uptake of ^18^F-FDG are imaging observations commonly identified in patients with CDC. When a renal tumor exhibits these imaging features, CDC may be suggested as a valuable differential diagnosis.

## Introduction

Collecting duct carcinoma (CDC) of the kidney, also known as Bellini duct carcinoma, is an extremely rare variant of renal cell carcinoma (RCC), accounting for 0.4–1.8% of all RCCs (1). In contrast to the considerably more common variants of RCC, arising from the convoluted tubules of the renal cortex, CDC is derived from the renal medulla, possibly from the distal collecting ducts of Bellini (1–3). Approximately four decades ago, Mancilla-Jimenez *et al*(4) first observed the atypical hyperplasia of the adjacent collecting ducts epithelium in three cases of papillary RCC. Therefore, the authors speculated that a few papillary RCCs may derive from the epithelium of the collecting ducts. Until 1979, the term Bellini duct carcinoma was presented by Cromie *et al*(5) It is worth noting that CDC has other synonyms besides Bellini duct carcinoma, including medullary renal carcinoma, distal nephron carcinoma and distal renal tubular carcinoma. In 1997, in accordance with the morphological aspect and chromosome of the primary renal cancer, five histologic types was defined in the Heidelberg classification (6), including the conventional, chromophobe, papillary, collecting duct and unclassifiable carcinoma. CDC is characterized by a tremendously aggressive phenotype. Patients with CDC usually have metastatic diseases at the time of presentation. Radical nephrectomy is the basis of therapy. Several systemic treatment protocols, including chemotherapy, radiotherapy and immunotherapy have been considered. However, these treatments do not produce a favorable response in the majority of CDC patients, and ~70% of patients succumb due to CDC progression within 2 years of diagnosis.

In general, CDC is considered to have a poor prognosis and early diagnosis is likely the only factor leading to a prolonged survival for patients (7). However, due to the rarity of this tumor and the lack of clinical awareness, no reliable diagnostic protocol has been established. To achieve an improved understanding of CDC and diagnosis, the present study analyzed the imaging features of six CDC patients treated in Jinling Hospital, Clinical school of Medical College, Nanjing University (Nanjing, China), between June 2007 and October 2012.

## Patients and methods

### Patient characteristics

The current retrospective study was approved by the institutional review board of Jinling Hospital, Clinical school of Medical College, Nanjing University and written informed consent forms were obtained from all patients.

In total, six patients (three males and three females; age range, 22–70 years; mean age, 46 years) with pathologically confirmed CDC of the kidney during the past five years were included.

The clinical information included the age, gender and clinical presentation of these patients. The radiological results available for analysis included non-contrast computed tomography (CT) in all six patients, contrast-enhanced CT in five patients, magnetic resonance (MR) urography in one patient, renal dynamic imaging and glomerular filtration rate (GFR) measuring in one patient and conventional whole-body ^18^F-fluorodeoxyglucose (FDG) positron emission tomography (PET)/CT in two patients.

### CT analysis

Abdominal CT was performed using a Siemens Somatom Emotion 6 or Somatom Definition (Siemens Healthcare, Erlangen, Germany), with the following scan parameters for imaging acquisition: 120–130 kVp, 110–340 mA, and a reconstruction thickness of 1–8 mm. Following the non-contrast CT scan, 100–120 ml IV contrast agent was injected into an antecubital vein at a rate of 3.0 ml/sec in five patients. Triphasic contrast-enhanced CT was performed, including arterial, nephrographic and excretory phases, with 25, 60 and 180 sec, respectively. A series of characteristic parameters describing the tumors consisted of the number of the tumors, tumor size, solid, cystic or complex mass, CT attenuation of the solid component, tumor location, inside features of the tumor (calcification, pseudocapsules and cystic components), degree and pattern of enhancement, metastatic lesions of the tumors (direct invasion to the renal pelvis and ureter, perinephric invading, region lymphadenopathies and distant metastases) and pattern of tumor growth.

The CT attenuation of the solid component was classified as high, equal or low compared with contralateral normal kidney. The location of tumor was classified as medullary, cortical or exophytic depending on the predominance. Medullary location was supported by intrusion of the renal pelvis, replacement of the renal sinus fat or distortion of the intrarenal collecting system. Cortical location was supported by a peripheral location of the tumor and contact with the outer renal capsule. An exophytic location was considered to be present when the major section of the tumor extended beyond the predicted renal confines. The presence of calcification was described on the non-contrast CT scan. A cystic component was considered to be present if a well-defined, liquid-like attenuation area was noted in the tumor.

In five cases where the contrast-enhanced CT was available, the degree and pattern of enhancement were determined on the nephrographic phase. The presence of vascular invasion was described on the contrast-enhanced CT scan and the presence of an infiltrative or expansile pattern of growth was defined by which pattern predominated in each case. On CT, infiltrative growth was characterized by poorly marginated borders between the tumor and normal renal parenchyma. On the contrary, expansible growth was characterized by well-defined bulging tumor margins that displaced the normal parenchyma.

Lymphadenopathy was defined when a lymph node was enlarged by >1 cm in diameter. Perinephric invading was defined when there was evidence of nodules with soft-tissue attenuation in the perinephric area and thickening of Gerota’s fascia. In addition, chest CT and cranial MR were performed in each patient to detect extra-abdominal metastatic lesions.

### MR urography analysis

MR urography was performed by a 3-Tesla scanner (Siemens Healthcare) using a torso phased array coil. Breath-hold, coronal thin slice and thick-slab T2-weighted single-shot fast spin-echo were obtained. Technical parameters for thin section T2-weighted single-shot fast spin-echo sequences were as follows: Repetition time (2,400 msec)/echo time (710 msec); 384×384 matrix; 1.5-mm section thickness; and 48-cm field of view. Technical parameters for the thick-slab T2-weighted sequences were as follows: 256×256 matrix; 5-mm thickness; and 40-cm field of view. The tumors that presented in the renal collecting system and ureter were evaluated.

### Single photon emission computed tomography (SPECT) analysis

SPECT (Siemens E.Cam; Siemens Healthcare) was used to perform renal dynamic imaging and the measurement of GFR. In total, 185 MBq ^99^Tc^m^-DTPA was used for the patient. The radioactivity (counts) of the pre-injection syringe containing ^99^Tc^m^-DTPA was determined at a distance of 20 cm from the detector for 60 sec. The patient consumed 300 ml of water prior to imaging and was then kept supine with the back facing the detector. The renal images were captured dynamically following a ‘bolus’ injection of 1 ml ^99^Tc^m^-DTPA (185 MBq). The acquisition conditions were as follows: Low-energy collimator; energy peak, 140 KeV; window width, 20%; and matrix, 128×128. In total, 20 frames of slow dynamic acquisition at a rate of one frame per 60 sec were collected following 30 frames of rapid dynamic acquisition at a rate of one frame per 2 sec. Once the images were captured, the radioactivity (counts) of the post-injection empty syringe was determined at a distance of 20 cm from the detector for 60 sec. GFR normalized to body surface area was calculated automatically from the renal dynamic images. The observations of renal dynamic imaging and GFR measuring were analyzed.

### PET/CT analysis

Conventional whole-body PET/CT was performed using a Siemens Biograph Sensation 16 (Siemens Healthcare). The patients were fasted for ≥6 h to maintain the blood glucose level at 3.9–6.1 mmol/l. A mean dose of 5.55 MBq/kg (0.15 mci/kg) of ^18^F-FDG was administrated intravenously to each patient. Imaging was initiated following an ^18^F-FDG uptake period of 60 min. Each patient underwent a total body scan that contained two steps of body and brain scanning. The non-contrast CT scan was performed immediately prior to the PET scan with a 16-slice multidetector spiral CT scanner. The CT results on the combined scanner were used for PET attenuation correction. CT, PET and PET/CT fused images were reconstructed in coronal, sagittal and transaxial projections on a computer screen with ordered subset expectation maximization iterative algorithm. All PET/CT images were interpreted using visualization and semi-quantitative analysis [maximum standardized uptake value (SUVmax)]. The SUVmax of each lesion, which was found by CT scanning or showed a high ^18^F-FDG uptake (SUVmax, >2.5), was measured and analyzed carefully.

### Surgical analysis

In total, five patients underwent nephrectomy and one patient underwent nephroureterectomy. The gross and microscopic features of the tumors were described by two pathologists. In addition, one patient underwent pleural biopsy and was diagnosed with multiple pleural metastases of CDC.

The time intervals between each examination and the surgery were <14 days. All images were retrospectively reviewed by three experienced radiologists, to reach a consensus in each patient.

## Results

### Clinical observations

The predominate manifestations that brought the patients to clinical attention included flank pain (n=4), fever (n=3), weight loss (n=3), gross hematuria (n=2), palpable mass (n=2) and chest pain (n=1).

### CT observations

**Figure 1:**
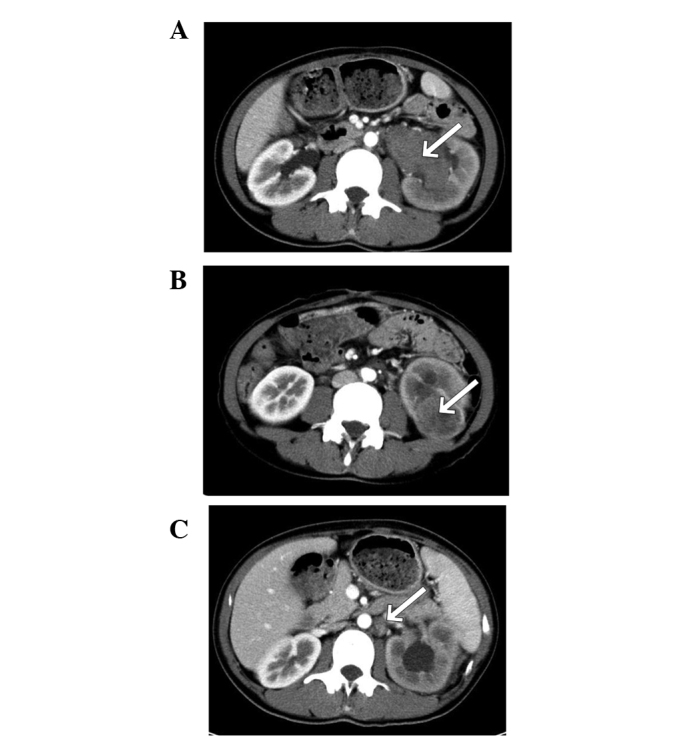
Imaging observations of patient 2. (A and B) Two tumors were identified in the left kidney. (C) Multiple lymph nodes were found in the renal hilum area, but no evidence of lymph nodes metastases was detected by pathological examination.

**Figure 2:**
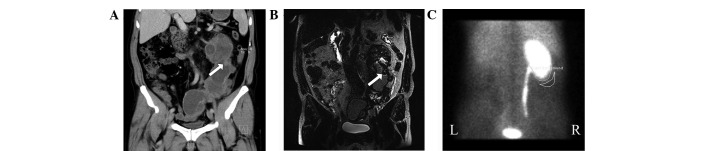
Imaging observations of patient 4. Coronal (A) contrast-enhanced computed tomography and (B) T2-weighted images showed the distention of the renal pelvis and ureter, as well as multiple nodular thickening of the wall of the ureter. (C) Fused image of the clearance phase of renal dynamic imaging failed to show the left kidney, which denoted that the renal function of the left kidney had been lost.

In total, seven tumors were found in the six cases, with two tumors detected in the left kidney of patient 2 (Fig. 1A and B). The longest diameter of the tumors ranged between 4.0 and 7.5 cm, and the mean size was 5.3 cm. In one case, the boundary of the tumor was not defined by CT scanning (patient 4; Fig. 2A); therefore, size was determined on the gross specimen. The tumors appeared solid (2/7) or complex (5/7) on CT. On non-contrast CT scanning, high, equal and low attenuation was observed in two, four and one tumors, respectively. In total, six tumors were located in medullary areas and only 1 tumor was found in the cortical location. A tiny calcification was present in only one tumor and cystic components were observed in five tumors, but no pseudocapsule was observed. Weak enhancements were observed in all six tumors examined with contrast-enhanced CT, and heterogeneous enhancements were also observed in the majority of these tumors with the exception of one tumor. An infiltrative pattern of tumor growth was present in five tumors, with an expansible appearance in the remaining two tumors.

Metastatic lesions were found in all six patients. Regional lymphadenopathies were observed in five cases, located in renal hilum and retroperitoneal areas. No evidence of lymph node metastases was shown in one of these five cases by pathology (patient 2; Fig. 1C), although multiple lymph nodes were found in the renal hilum area. Perinephric invading was observed in one case and direct invasion of the renal pelvis and ureter were observed in two cases. Distention of the renal pelvis and almost total ureter, multiple nodular thickening on the wall of the ureter, extensive destruction of the calyceal structure and hydronephrosis and hydroureterosis existed in one patient (patient 4; Fig. 2A), in which pyonephrosis and inflammatory infiltrates were found. Renal or inferior vein tumor thrombus were not observed. Multiple pleural metastases were detected by chest CT in one patient (patient 6; Table I).

### MR urography observations

The MR urography was performed on only one patient (patient 4). Similar to the CT observations, the boundary of the tumor was not clearly defined (Fig. 2B). However, the destruction of the renal pelvis and wall of the ureter and the extent of the hydronephrosis and hydroureterosis were shown more distinctly.

### Renal dynamic imaging and measurement of GFR

The renal dynamic imaging was performed on only one patient (patient 4). In these images, the left kidney was not detected. This denoted that the renal function of the left kidney had been lost (Fig. 2C). However, the renal function of the right kidney increased complementally and the normalized GFR was 121.5 ml/min.

### PET/CT observations

**Figure 3:**
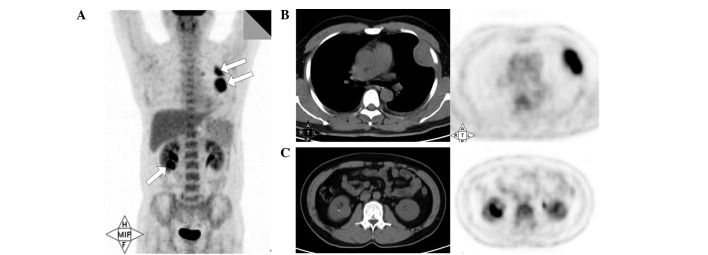
Imaging observations of patient 6. (A) Maximum intensity projection and (B and C) positron emmission tomography/computed tomography images showed a renal mass in the right kidney and multiple pleural metastases in the left thoracic cavity. A marked uptake of ^18^F-FDG was observed in each lesion.

The ^18^F-FDG PET/CT was performed on two patients (patients 1 and 6). The malignant lesions, including primary tumors, regional lymphadenopathies and distant metastases, found by PET/CT were consistent with those detected by pre- and post-contrast CT scanning. In addition, the SUVmax was >2.5 in each lesion (Fig. 3).

## Discussion

CDC is a rare epithelial neoplasm in the kidney and is recognized as a distinct entity in the 2004 World Health Organization classification (8–10). Tokuda *et al*(11) reported the largest series of exclusive CDC cases in 2006, which were collected throughout Japan across 66 institutions. Of these, the median age was 58.2 years and males comprised of 71.6% of the patient population. However, this demographic profile also applies to the more common RCCs and is not an effective differential point.

Clinical manifestations of CDC in the present study were consistent with those of more common RCCs, including flank pain, hematuria and palpable mass. Constitutional symptoms, including fever and weight loss, are also common, but no particular paraneoplastic syndrome was observed (3,12). In addition, one of the patients showed evident chest pain, which may have been caused by the pleural metastasis.

To date, the imaging features of CDC are not well characterized, since only case reports or studies involving small numbers of patients have been published (1,3,12–18). Previously, Pickhardt *et al* (§1) described the radiological observations of 17 patients with histopathologically confirmed CDC. In the authors’ series, medullary involvement (94%) and an infiltrative appearance (65%) were common observations of CDC, and a cystic component (35%) and calcification were frequently identified within the tumors. An additional study by Yoon *et al*(14) has reported the largest radiological series in the literature. In the total 18 cases, the authors found that medullary location (94%), weak (69%) and heterogeneous (85%) enhancement, involvement of the renal sinus (94%), infiltrative growth (67%), preserved renal contour (61%) and a cystic component (50%) were CT observations frequently identified in patients with CDC. At the same time, regional lymphadenopathy, perinephric stranding, vascular invasion and distant metastases were observed in 56, 56, 28 and 33% of the patients.

In the present study, a total of six patients, including monofocal and multifocal cases, exhibited seven tumors. In general, the tumors presented as solid or complex solid and cystic on CT. Renal medullary involvement was the most common observation of CDC identified in six tumors. In contrast to the more common RCCs, weak and heterogeneous enhancement were the general appearance in contrast-enhanced CT scans of the CDCs. Calcification, cystic components and pseudocapsule were observed in 1, 5 and 0 tumors, respectively. An infiltrative pattern of tumor growth was present in the majority of the tumors. In addition, local, lymphatic or hematogenous spreading was noted in all CDCs, which predicted an aggressive biological behavior and a poor long-term prognosis. Regional lymphadenopathies were observed in five cases, but no lymph node metastases were detected in one of these cases. This demonstrated that lymphadenopathies are not necessarily caused by lymph nodes metastases. Pyonephrosis and inflammatory infiltrates were detected in one case, which may have been caused by the secondary upper urinary tract obstruction.

MR urography is an evolving member of the urologic imaging armamentarium. It evaluates the renal parenchyma and surrounding structures besides the renal collecting systems, ureters and bladder (19–23). The two most common sequences used in MRU are a heavily T2-weighted hydrographic sequence without contrast material and a T1-spoiled GRE sequence during the excretory phase following gadolinium based contrast administration. Previous studies have shown that MRU detects tumors of the upper urinary tract with high accuracy using T2-weighted MRU only (22,23). In the current study, the extent and the surrounding structures of the tumor were shown more clearly by MR urography. From these images, the doctors of urinary surgery determined that the patient undergo nephroureterectomy rather than nephrectomy.

The GFR, the plasma volume filtering through the glomerulus per minute, is a significant index for the assessment of the renal function. Currently, renal dynamic imaging is widely used in clinical practice to calculate the GFR (24–26). In the present study, the purpose of this examination was to evaluate the renal function of the healthy kidney. The renal function of the involved kidney was virtually lost, at the same time, the renal function of the healthy kidney increased complimentally and the normalized GFR was 121.5 ml/min. Therefore, the renal insufficiency was not likely to occur following nephroureterectomy.

The most commonly used radionuclide in PET is ^18^F-FDG, which is the analog of D-glucose. Malignant tumors are more metabolically active than their normal surrounding tissues and are likely to uptake more ^18^F-FDG. This high concentration of the radiotracer produces a detectable signal greater than the background, allowing the isolation of tumor location. However, in previous studies, the detection of common RCCs with PET scanning has been hampered by the fact that ^18^F-FDG is excreted via the kidneys (27–29). Due to the rarity of the CDC, few previous studies have analyzed the appearances of PET imaging (1,30). In a previous study by Ye *et al*(30), a CDC, with the longest diameter of 4.6 cm and SUVmax of 7, located in the right kidney was reported. Yang *et al*(1) also described PET/CT images of a CDC with distal ureteral seeding metastasis. However, in this study, only faint nodular ^18^F-FDG uptake was observed in the primary tumor. In the current series, PET/CT scanning was performed on two patients and an evidently high uptake of ^18^F-FDG was observed in each tumor. In addition, the PET/CT images showed a marked ^18^F-FDG uptake in the regional lymphadenopathies and pleural metastases, which is consistent with the study by Yang *et al*(1).

The differential diagnoses for CDC include renal clear cell carcinoma, invasive transitional cell or squamous cell carcinoma, renal lymphoma and metastases, mesoblastic nephroma, renal medullary carcinoma and bacterial pyelonephritis (12,14). As the most common renal malignant tumor, renal clear cell carcinoma usually locates in the renal cortex with a pseudocapsule and is hypervascular, in contradistinction to CDC. The invasive transitional cell or squamous cell carcinoma locates in the pelvis and ureter, but usually invades to the renal medulla and is hypovascular. It is difficult to distinguish these two types of cancer from CDC. Renal lymphoma locates in the renal medulla, but rarely shows cystic components or calcification prior to treatment. Renal metastatic lesion, usually from a primary lung cancer, is typically multiple and bilateral. Mesoblastic nephroma often occurs in infancy and rarely in adults. Renal medullary carcinoma is an aggressive malignancy that is closely associated with sickle cell trait. Bacterial pyelonephritis is distinguished on a clinical basis. However, all of these entities demonstrate significant overlap on imaging observations.

To date, few studies have analyzed the imaging characteristics of CDC. In addition to confirming observations reported by previous studies, the current study identified several additional features regarding the imaging appearance of CDC. Firstly, to the best of our knowledge, the present study is the first to report multifocal CDC in the same kidney. It demonstrated that multifocus may occasionally be observed in the patients of CDC, although the majority of patients were monofocal. Secondly, the widespread infiltration of renal pelvis and ureter was observable. Although a few cases of ureteral metastasis have been reported in the previous literature, the extent of the malignant lesions has been shorter than in the present study (1,18). Thirdly, the current study suggested that PET/CT scanning may provide additional information for detecting and grading CDC, due to the high uptake of the ^18^F-FDG.

There were several limitations of the present study. Firstly, the imaging results obtained of CDC were too small, particularly for MRU, renal dynamic imaging and PET/CT. Therefore, the study was limited in terms of the statistical analysis of imaging observations. Secondly, not all enlarged lymph nodes obtained reliable pathological results, due to the difficulties of the surgery and, finally, specific imaging features of CDC were not obtained. Certain common imaging observations may have appeared for the other subtypes of RCC; therefore, future studies with large numbers of patients is necessary.

The informative imaging observations of the CDC obtained in the present study include monofocal or multifocal lesions, solid or complex solid and cystic mass, medullary location, weak and heterogeneous enhancement, infiltrative growth, a cystic component, damage of renal function in the involved kidney and a marked uptake of ^18^F-FDG. Furthermore, direct invasion of the perirenal fascia, renal pelvis and ureter, regional lymph nodes and distant metastases were observed. However, these imaging features may be observed in other more common renal diseases as aforementioned. Therefore, these imaging appearances are non-specific and may not allow CDC to be reliably distinguished from these diseases. However, when a renal tumor exhibits these imaging observations, CDC may be suggested as a valuable differential diagnosis.

## Figures and Tables

**Table I:** CT observations of six CDCs.

Patient no.	Age, years/gender	Longest diameter, cm	CT attenuation	Location	Pattern of enhancement	Inside features	Pattern of tumor growth	Metastatic lesions
1	70/F	6.5	Equal	Medullary	Weak and homogeneous	None	Infiltrative	Multiple lymph node metastases in renal hilum area
2^a^	22/F	4.0/4.0	High/high	Medullary/cortical	Weak and heterogeneous/weak and heterogeneous	Cystic component/cystic component	Infiltrative/expansible	Direct invasion to the renal pelvis and multiple enlarged lymph nodes in renal hilum^b^
3^c^	53/M	6.0	Low	Medullary	-	None	Infiltrative	Direct invasion to the perirenal fat and multiple lymph node metastases in renal hilum and retroperitoneal areas
4^d^	50/M	7.5	Equal	Medullary	Weak and heterogeneous	Cystic component	Infiltrative	Direct invasion to the renal pelvis and ureter
5	30/F	5.0	Equal	Medullary	Weak and heterogeneous	Cystic component	Infiltrative	Lymph node metastasis in renal hilum area
6	46/M	4.0	Equal	Medullary	Weak and heterogeneous	Cystic component and calcification	Expansible	Lymph node metastasis in renal hilum area and multiple pleural metastases

a Two tumors identified;

b no evidence of tumor involvement was detected by pathology;

c non-contrast CT scanning only;

d boundary of the tumor was not defined clearly by CT scanning.

CT, computed tomography; CDCs, collecting duct carcinomas; F, female; M, male.
